# Hepatic artery infusion chemotherapy combined with camrelizumab plus rivoceranib for hepatocellular carcinoma with portal vein tumor thrombosis: a multicenter propensity score-matching analysis

**DOI:** 10.1007/s12072-024-10672-8

**Published:** 2024-05-08

**Authors:** Yangyang Li, Jiandong Guo, Wendao Liu, Huajin Pang, Yipei Song, Siyi Wu, Fengtao Zhang, Dong Yan, Junwei Chen, Chao An, Chengzhi Li

**Affiliations:** 1 Department of Interventional Radiology and Vascular Surgery, The First Affiliated Hospital of Jinan University, No.613 of West Huangpu Avenue, Guangzhou, Guangdong, 510630 People’s Republic of China; 2 Department of Interventional Therapy, Guangdong Provincial Hospital of Chinese Medicine and Guangdong Provincial Academy of Chinese Medical Sciences, No. 111 Dade Road, 510080 Guangzhou, Guangdong People’s Republic of China; 3 Division of Vascular and Interventional Radiology, Department of General Surgery, Nanfang Hospital, Southern Medical University, Guangzhou, Guangdong 510515 People’s Republic of China; 4 Department of Radiology, The Second Affiliated Hospital of Nanchang University, Nanchang, China; 5 Department of Interventional Therapy, Huazhong University of Science and Technology Union Shenzhen Hospital, Shenzhen, Guangdong China; 6 Department of Oncology, Beijing Luhe Hospital, Capital Medical University, Beijing, 101199 China; 7 Department of Interventional Radiology, The Third Affiliated Hospital of Sun Yat-Sen University, Tianhe Road 600#, Tianhe District, Guangzhou, 510630 Guangdong, People’s Republic of China; 8 Department of Minimal Invasive Intervention, Sun Yat-Sen University Cancer CenterState Key Laboratory of Oncology in South ChinaCollaborative Innovation Center for Cancer Medicine, 651, Dongfeng East Road, Guangzhou, People’s Republic of China

**Keywords:** Hepatocellular carcinoma, Hepatic artery infusion chemotherapy, Camrelizumab, Rivoceranib, Portal vein tumor thrombosis, Propensity score matching

## Abstract

**Background:**

Portal vein tumor thrombosis (PVTT) signifies late-stage hepatocellular carcinoma (HCC) with high-risk progression and poor prognosis. As a standard treatment, sorafenib monotherapy has limited the efficacy in managing HCC with PVTT. Currently, both hepatic arterial infusion chemotherapy (HAIC) and the combination of camrelizumab and rivoceranib have shown favorable survival benefits for advanced HCC, surpassing the standard sorafenib treatment. In this study, we investigate the safety and efficacy of HAIC combined with camrelizumab and rivoceranib in treating HCC patients with PVTT.

**Methods:**

From January 2020 to December 2021, HCC patients with PVTT, who received either a triple regime of HAIC combined with camrelizumab and rivoceranib or a dual regime of camrelizumab and rivoceranib as their first-line treatment, were reviewed for eligibility at four hospital centers in China. To balance any intergroup differences, propensity score matching (PSM) was applied. The aim of this study is to compare the efficacy of the dual and triple combination treatment regimens based on survival prognosis and tumor response and evaluate the safety based on the occurrence of adverse reactions.

**Result:**

In this study, a total of 411 patients who received either the triple treatment regime (HAIC combined with camrelizumab plus rivoceranib, referred to as the HAICCR group, *n* = 292) or the dual treatment regime (camrelizumab combined with rivoceranib, referred to as the CR group, *n* = 119) between January 2020 and December 2021 were included. The results showed that the HAICCR group exhibited significantly better overall survival (mOS: 19.60 months vs. 11.50 months, *p* < 0.0001) and progression-free survival (mPFS: 10.0 months vs. 5.6 months, *p* < 0.0001) compared to the CR group in the overall cohort. Moreover, the HAICCR group also had a significantly higher ORR (objective response rate, 55.5% vs. 42.0%, *p* = 0.013) and DCR (disease control rate, 89.0% vs. 79.0%) compared to the CR group. After PSM, a final matched cohort of 83 pairs was obtained, and the survival benefits were consistent in this cohort as well (mOS: 18.70 months vs. 11.0 months, *p* < 0.0001; mPFS: 10.0 months vs. 5.6 months, *p* < 0.0001). However, there was no significant difference in the ORR between the triple and dual combination regimes. Univariate and multivariate analysis showed that CTP (Child–Turcotte–Pugh) stage, ALBI (albumin–bilirubin index) grade, tumor number, and treatment regime were significant risk factors affecting overall survival, while AFP (α-fetoprotein) level, tumor number, metastasis, and treatment regime were significant risk factors affecting progression-free survival. As for safety, hypertension and hand–foot syndrome were the two most common adverse reactions in both groups, with no significant difference in the occurrence of adverse reactions between the two groups (*p* < 0.05).

**Conclusion:**

In the context of advanced HCC patients with PVTT, the combination regime of HAIC and camrelizumab plus rivoceranib demonstrates more excellent capacity for prolonging survival and offers a well-tolerated safety compared to the CR dual therapy approach. This triple regime represents a therapeutic modality of broad prospects and vast potential for HCC patients with PVTT.

## Introduction

Hepatocellular carcinoma (HCC) is a prominent contributor to malignancy-related fatalities worldwide, the tumor occurrence and progression are frequently accompanied by the simultaneous presence of tricky sequelae [1]. Portal vein tumor thrombosis (PVTT) is one of the common complications of HCC, with an incidence ranging from approximately 44 to 62% [2, 3]. The involvement of the portal vein often presents the thorny predicament of augmented tumor burden and progressive deterioration in liver function resulting from widespread intrahepatic metastasis and portal hypertension, which consequently leads to unsatisfactory treatment outcomes with a median overall survival of 2.7–4.0 months under the support of placebo only [4]. Unfortunately, as the recommended standard treatment for HCC with PVTT, the intervention of sorafenib can only provide a modest extension of survival (nearly 2 months) [5–7].

Currently, the combination of anti-angiogenic and immunotherapy modalities is widely regarded as a more advantageous treatment approach for late-stage HCC with or without PVTT than oral sorafenib. In the prior two randomized controlled trials (RCT), IMBRAVE-150 and KEYNOTE-524 [8, 9], exploring the integration of anti-angiogenic therapy and the blockade of the PD-1/PD-L1 (programmed cell death protein 1/programmed death ligand (1) pathway, has shown a promising landscape. Given the compelling results from previous clinical research, both camrelizumab (a selective humanized IgG4 (immunoglobulin G4) monoclonal antibody) and rivoceranib (also known as apatinib, a highly selective VEGFR-2 (vascular endothelial growth factor receptor (2) inhibitor) have exhibited notable efficacy in tumor management and controllable adverse reactions as second-line treatments for pretreated advanced HCC patients [10, 11]. As reported in the ASCO (American Society of Clinical Oncology) 2022 meeting, camrelizumab combined with rivoceranib demonstrated favorable anti-tumor efficacy and satisfactory survival outcomes across a spectrum of solid malignancies [12–14]. Recently, a nonrandomized open-label phase II Trial (RESCUE) has initially confirmed that camrelizumab plus rivoceranib holds promise as a potential treatment option for advanced HCC in both the first-line and second-line contexts [15]. Consequently, the CARES-310 trial achieved encouraging survival benefits with a remarkable median overall survival of 22.1 months and received an advantageous survival in the PVTT subgroup [16]. Based on these research findings, the National Medical Products Administration (NMPA) in China has granted approval for camrelizumab–rivoceranib as a first-line treatment option for advanced HCC [17]. Moreover, the integration of camrelizumab and rivoceranib may also possess extensive potential in managing PVTT as reported in a recent retrospective study.

Transarterial chemoembolization (TACE) is universally recognized as the standard therapy for advanced HCC; however, its efficacy tends to be limited when dealing with HCC patients complicated by the presence of PVTT [18]. Hepatic arterial infusion chemotherapy (HAIC) is a catheter-based local treatment that differs from traditional systemic chemotherapy by delivering high drug concentrations locally while minimizing systemic side effects. A recent phase III randomized trial has provided evidence that HAIC is a superior choice to TACE for HCC patients with high tumor burden [19]. HAIC monotherapy and combination therapy have been recommended as the optimal strategies for advanced HCC with major PVTT according to China guidelines [20]. On the other hand, the advantage of HAIC in enhancing systemic treatment efficacy and its modest adverse effects for HCC patients with or without PVTT has been extensively corroborated in several retrospective research [21–23]. Therefore, the integration of HAIC and targeted-plus immunotherapy is anticipated to confer additional advancements in terms of survival for HCC with PVTT.

To date, the efficacy and safety of the triple combination regime of HAIC and camrelizumab plus rivoceranib in HCC with PVTT remains an unmet area. Therefore, this multicenter retrospective study aims to investigate the survival benefits of combining HAIC with camrelizumab and rivoceranib to enhance the therapeutic efficacy against PVTT.

## Methods

### Study population

From January 2020 to December 2021, a qualification assessment was conducted on 592 patients diagnosed with advanced HCC and PVTT in four Chinese hospital centers treated with either a triplet regimen of HAIC and camrelizumab–rivoceranib (HAICCR group) or a dual regime of camrelizumab and rivoceranib (CR group) as first-line treatment. The inclusion criteria: HCC was diagnosed pathologically or by intravenous contrast-enhanced computed tomography (CT) or magnetic resonance imaging (MRI), according to the American Association for the Study of Liver Diseases (ASSLD) guideline; aged 18 or above; Eastern Cooperative Oncology Group Performance Status (ECOG PS) score of 0–1; liver function classified as albumin–bilirubin index (ALBI) grade 1–2; tumor staging as Barcelona Clinic Liver Cancer (BCLC) C stage with confirmed portal vein involvement based on imaging data; receiving at least two cycles or more of combination therapy. The exclusion criteria were as follows: loss to follow-up; missing clinical data; prior receipt of other tumor-related treatments before enrollment; presence of other malignant tumors. The radiological imaging evaluation included abdominal contrast-enhanced CT/MRI examination within 1 week before treatment.

The institutional review committees at each center received ethical approval for this retrospective multicenter study. The present investigation adhered to the principles outlined in the Declaration of Helsinki. All patients included in this study provided informed consent for the treatment.

### Treatment procedure

All enrolled patients receiving the dual regimen were administered intravenous camrelizumab (with a dosage of 200 mg for patients weighing ≥ 50 kg or 3 mg/kg for patients weighing < 50 kg) every 14 days, in combination with oral rivoceranib (with a daily dosage of 250 mg) in treatment cycles of 28 days. The treatment was discontinued in the event of withdrawal of informed consent, intolerable adverse events, and disease progression. In the event of grade 3–4 TRAEs, camrelizumab and rivoceranib treatment was interrupted or the dosage of rivoceranib (250 mg/day for 5 days with 2 days off, or 7 days on with 7 days off) was reduced. The discontinuation of camrelizumab and/or rivoceranib in cases of disease progression and uncontrollable TRAEs was appropriate and alternative second-line treatment options can be considered for continuing follow-up.

For the triplet therapy group, hepatic artery catheterization is performed by two experienced interventional radiologists under digital subtraction angiography (DSA) guidance. The procedure is as follows: under local anesthesia, the modified Seldinger method was performed to puncture the femoral artery and the 5 F vascular sheath was sequentially inserted. Then, the 5-Fr Yashiro catheter (Terumo, Tokyo, Japan) was, respectively, inserted into the celiac trunk and superior mesenteric artery for angiography. After the tumor supply artery was clarified by radiography, the 2.7 F microcatheter (ASAHI, Tokyo, Japan) was coaxially inserted and super-selected to the tumor blood supply branch artery, and the microcatheter head end position was determined by micro-ductal angiography. After the microcatheter pathway was constructed, a modified FOLFOX6 (mFOLFOX6) protocol including oxaliplatin at a dose of 85 mg/m^2^ via intravenous 2 h drip on day 1, calcium folinate at a dose of 400 mg/m^2^ intravenous 2 h drip on day 1, and fluorouracil at a dose of 400 mg/m^2^ intravenous injection on day 1, followed by a continuous infusion of 2400 mg/m^2^ over 46 h was delivered through a microcatheter. HAIC dosage was reduced or interrupted according to the grades 3–4 adverse reactions. HAIC dose reduction is defined as decreasing the dose of 5FU to 300 mg/m^2^ bolus and 1800 mg/m^2^. HAIC interruption is defined as delaying the HAIC treatment cycle to the next one. For persistent grades 3–4 adverse reactions, HAIC was discontinued. Evaluations of therapeutic efficacy were conducted through periodic reviews of imaging data at intervals of 2 months after each treatment cycle.

### Survival outcomes and tumor response

Overall survival (OS, which was defined as the time starting from the initiation of treatment until either death occurred from any cause or the most recent follow-up assessment was conducted) and progression-free survival (PFS, which was defined as the interval from the initiation of tumor treatment to disease progression, as evaluated based on the mRECIST 1.1 criteria [24]) was measured and compared as the primary end point. Additionally, the efficacy of treatment was assessed using the mRECIST 1.1 criteria, which classified responses as complete response (CR), partial response (PR), stable disease (SD), or progressive disease (PD). Two experienced radiologists independently conducted all radiographic evaluations of tumor response. Objective response rate (ORR), which represented the proportion of patients who achieved a tumor response categorized as CR or PR, was considered the secondary end point, and the disease control rate (DCR, defined as the percentage of patients who achieved a tumor response categorized as CR, PR, or SD) was counted as the third end point. Furthermore, treatment-related adverse events (TAREs) were evaluated following the Common Terminology Criteria for Adverse Events, version 5.0.

### Data collection and follow-up protocol

The baseline clinical characteristics assessed in this study included gender, age, ECOG PS score, presence or absence of hepatitis B virus infection (HBV), hepatitis C virus infection (HCV), CTP stage, TBIL (total bilirubin), ALB (albumin), ALBI grade (calculated by: linear predictor = (log^10^ bilirubin mmol/L × 0.66) + (albumin g/L × -0.085)), BCLC stage, α-fetoprotein (AFP) level, largest tumor diameter, tumor number, and the presence or absence of metastasis. Radiological evaluations of the enhanced CT or MRI images were conducted every 4 weeks by two experienced radiologists in the first four cycles and every 8 weeks scans were reviewed afterward. Laboratory tests including rechecking tumor markers and liver function evaluation on the 3rd day after each treatment cycle were performed for efficacy and safety assessment.

### Propensity score matching (PSM)

The 1:1 PSM method was employed to balance the baseline differences between groups. The matching tolerance was adjusted to 0.02. The covariates included age, gender, ECOG PS score, CTP, ALBI grade, HBV, tumor size, tumor number, metastasis, and AFP level.

### Statistical analysis

The study analysis was conducted using R software (Rstudio version 4.2.2). Mean ± standard deviation was used to present continuous variables that followed a normal distribution, which were analyzed using the Student's *t* test. Otherwise, the median was used for representation and was analyzed utilizing the Mann–Whitney *U* test. Categorical variables were presented as percentages and analyzed using either the Chi-square test or Fisher’s exact test. The overall survival (OS) and progression-free survival (PFS) curves between groups in both the overall and matched cohort were estimated using the Kaplan–Meier method. To identify independent risk factors influencing OS and PFS, a Cox proportional hazards regression model was utilized. Statistical difference was determined with a two-tailed *p* value < 0.05.

## Results

### Patient characteristics

**Fig. 1:**
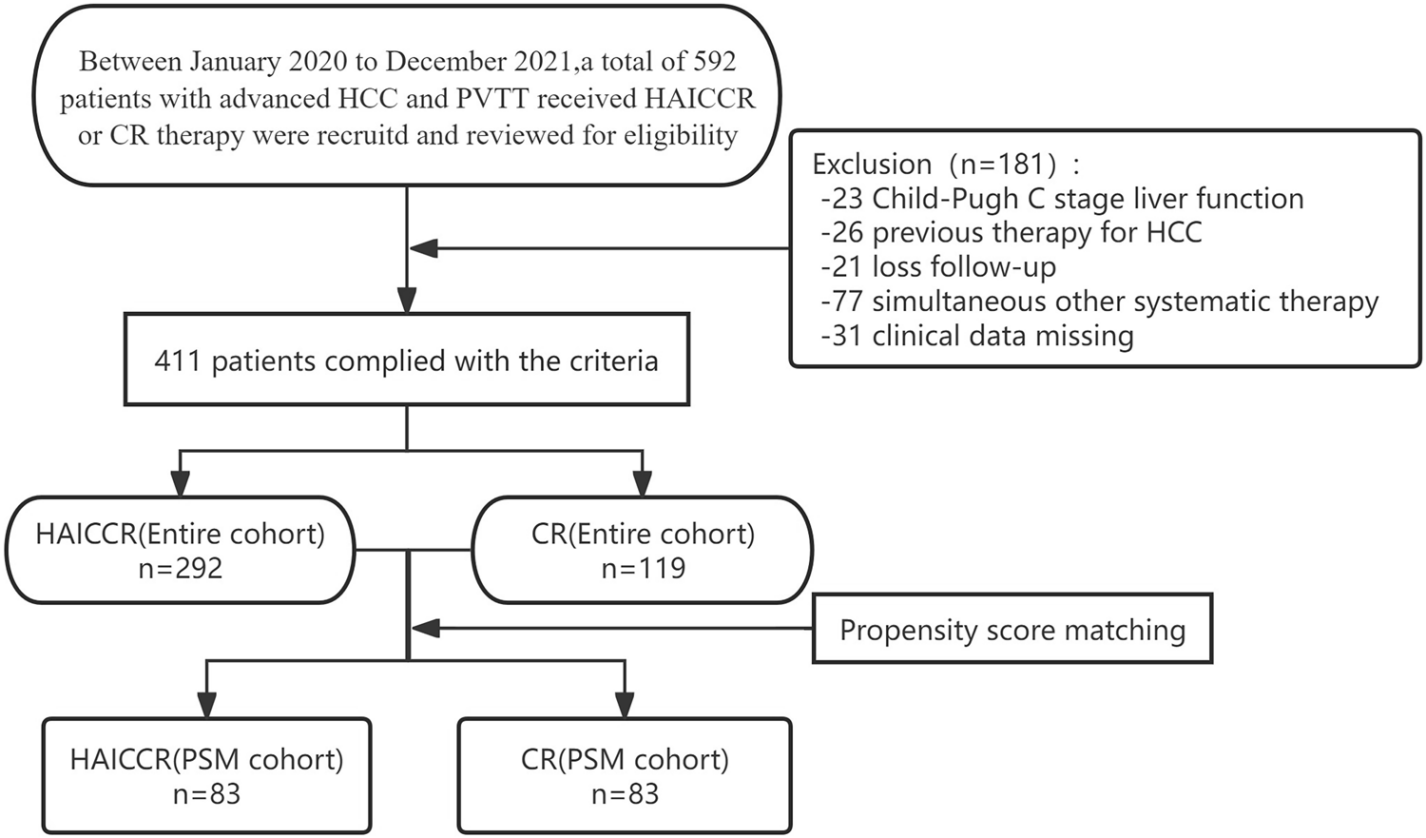
Flowchart of the patient selection process for this study

Through eligibility review, 292 HCC patients with PVTT who were treated with a first-line treatment of HAIC combined with camrelizumab and rivoceranib in a triplet regimen, as well as 119 patients who received a first-line treatment of camrelizumab combined with rivoceranib in a dual regimen, met the criteria and were eventually recruited in this study. The baseline characteristics and patient selection flowchart are illustrated in Table 1 and Fig. 1, respectively. The median age in the HAICCR group was 51 years and 267 of 292 (91.4%) male patients were included, while the CR group had a median age of 53 years with 111 out of 119 (93.3%) male patients. Out of the 292 patients in the HAICCR group, 271 (92.8%) were found to be infected with HBV. Similarly, among the 119 patients in the CR group, 111 (93.3%) were diagnosed with HBV infection. The median tumor diameter was 9.90 cm and 10.85 cm in the CR group and HAICCR group. In the overall cohort, the CR group had a greater prevalence of patients with an ECOG PS score of 1 and the presence of extrahepatic metastasis. After PSM adjustment, the differences between the groups mentioned above were eliminated, resulting in a final PSM cohort of 83 patient pairs.

**Table 1:** Baseline characteristics of enrolled patients in the entire cohort and the PSM cohort

	Entire cohort			PSM cohort		
	HAICCR group (*n* = 292)	CR group (*n* = 119)	*p* value	HAICCR group (*n* = 83)	CR group (*n* = 83)	*p* value
Gender			0.673			1.000
Male	267 (91.4)	111 (93.3)		76 (91.6)	77 (92.8)	
Female	25 (8.6)	8 (6.7)		7 (8.4)	6 (7.2)	
Age (median, IQR)	51.0 (43.0–58.0)	53.0 (45.0–58.0)	0. 453	52.0 (46.0–58.0)	51.0 (44.0–58.0)	0.764
≤ 65y	268 (91.8)	112 (94.1)		76 (91.6)	78 (94.0)	
> 65y	24 (8.2)	7 (5.9)		7 (8.4)	5 (6.0)	
ECOG			< 0.001			1.000
0	288 (98.6)	95 (79.8)		81(97.6)	82 (98.8)	
1	4 (1.4)	24 (20.2)		2 (2.4)	1 (1.2)	
Etiology			0.531			0.755
HBV	271 (92.8%)	111 (93.3)		77 (91.6)	78 (94.0)	
HCV	3 (1.0%)	0 (0%)		0 (0%)	0 (0%)	
None	18 (7.2%)	8 (6.7)		6 (8.4)	5 (6.0)	
CTP class			0.976			
A	267 (91.4)	108(90.8)		80(96.4)	75 (90.4)	0.212
B	25(8.6)	11(9.2)		3(3.6)	8(9.6)	
ALBI grade			0.535			0.755
1	149 (51.0)	56 (47.1)		36 (43.4)	39 (47.0)	
2	143 (49.0)	63 (52.9)		47(56.6)	44 (53.0)	
AFP			<0.001			0.876
≤ 400 ng/L	102 (34.9)	77 (64.7)		46 (55.4)	44 (53.0)	
>400 ng/L						
BCLC stage			1.000			1.000
A	0 (0)	0 (0)		0 (0)	0 (0)	
B	0 (0)	0 (0)		0(0)	0(0)	
C	292 (100)	119 (100)		83 (100)	83 (100)	
Tumor size			1.000			0.119
< 7cm	106 (36.3)	43 (36.1)		28 (33.7)	18 (21.7)	
≥ 7cm	186 (63.7)	76 (63.9)		55 (66.3)	65 (78.3)	
Tumor number			0.002			0.347
1–3	110 (37.7)	63 (52.9)		32 (38.6)	39 (47.0)	
> 3	182 (62.3)	56 (47.1)		51 (61.4)	44 (53.0)	
Metastasis			0.004			
Presence	158 (54.1)	46 (61.3)		51 (81.5)	41 (84.0)	0.160
Absence	134 (45.9)	73 (38.7)		32 (18.5)	42 (16.0)	

Data represent as *n*(%)

*PSM* propensity score matching, *ECOG* Eastern Cooperative Oncology Group Score, *HBV* hepatitis B virus, *CTP *Child–Turcotte–Pugh, *ALBI* albumin–bilirubin, *BCLC* Barcelona Clinic Liver Cancer,* AFP* α-fetoprotein, *HAICCR* hepatic artery infusion chemotherapy plus camrelizumab and rivoceranib, *CR* camrelizumab combined with rivoceranib

### Comparison of survival outcomes

**Fig. 2:**
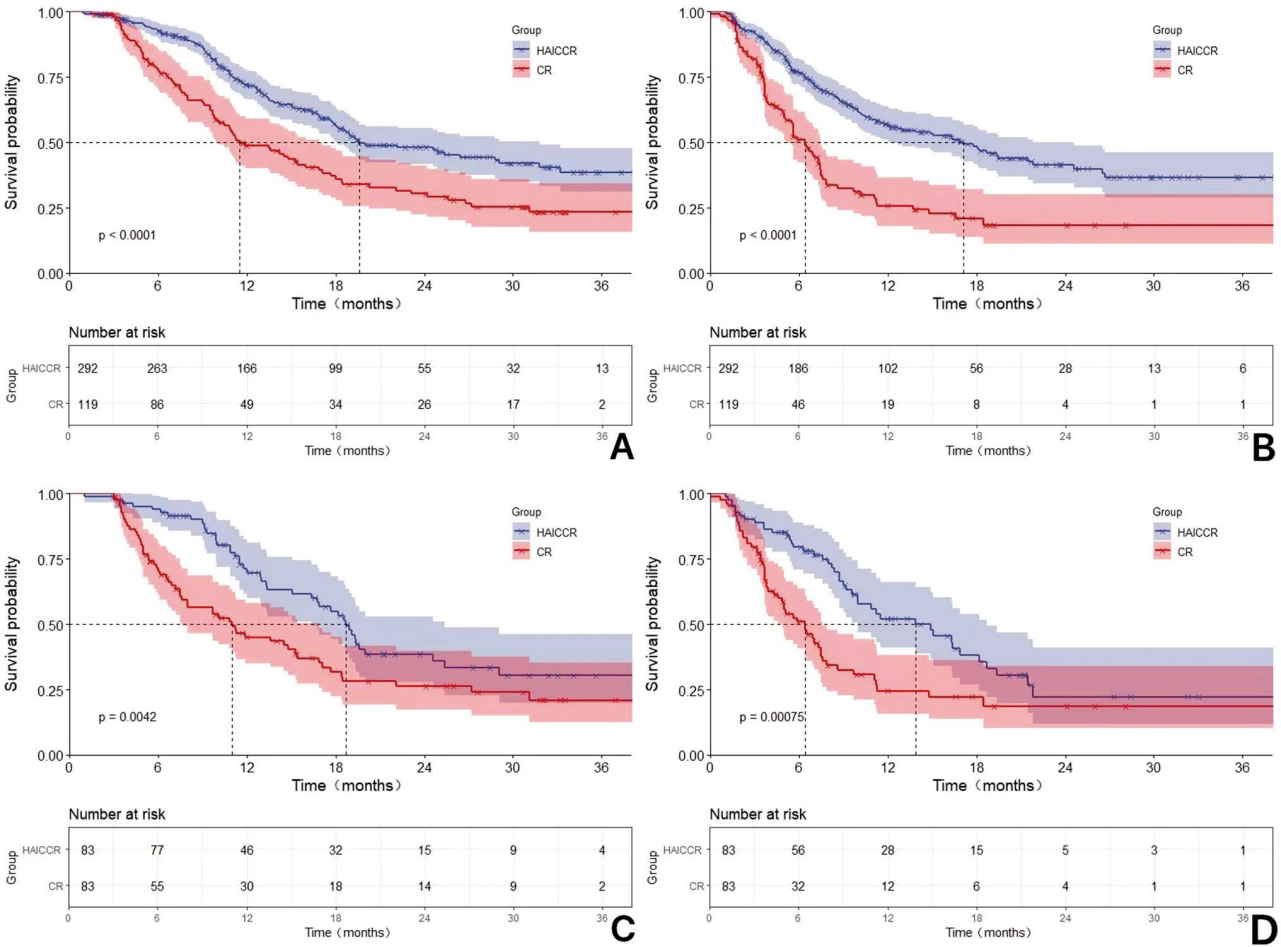
The Kaplan–Meier survival curves for the HAICCR group and the CR group with or without propensity score-matching (PSM) adjustment. (A) The Kaplan–Meier curves comparing the overall survival between the HAICCR group and the CR group without PSM-adjusted. (B) The Kaplan–Meier curves comparing the overall survival between the HAICCR group and the CR group without PSM-adjusted. (C). Comparison of PSM-adjusted overall survival between the HAICCR group and CR groups. (D) Comparison of PSM-adjusted progression-free survival between the HAICCR group and CR groups

**Fig. 3:**
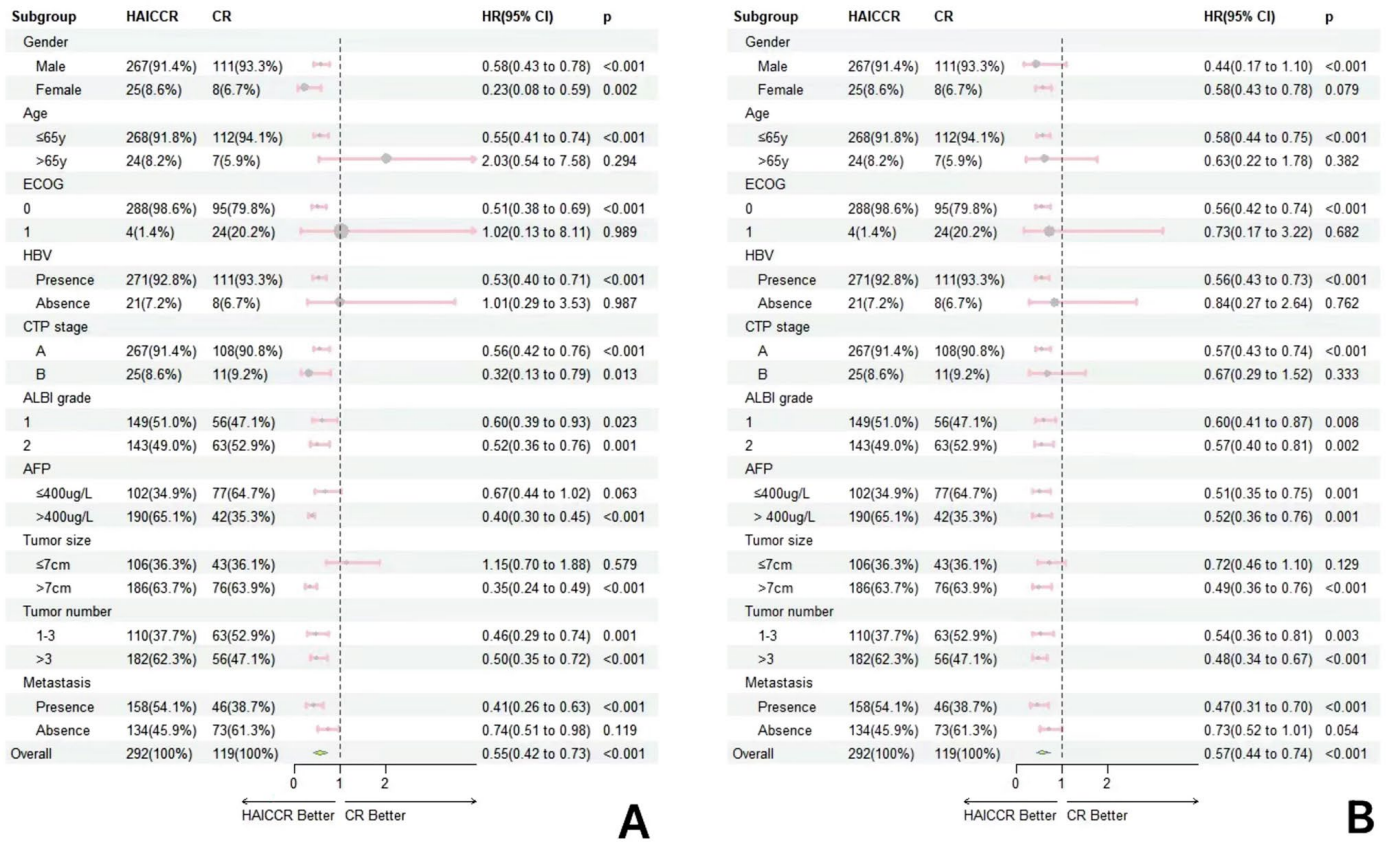
Forest plot based on overall survival(A) and progression-free survival(B) of each subgroup

The median follow-up time was 19.7 months. In comparison to the median OS of 11.5 months (HR: 1.63, 95% CI 8.31–14.70) and the median PFS of 5.6 months (HR: 0.92, 95% CI 8.21–11.79) in the dual regimen group, patients in the triple regimen group achieved an encouraging median OS of 19.6 months (HR: 0.56, 95% CI 4.49–6.71) and median PFS of 10.0 months (HR: 0.92, 95 % CI 8.21–11.79). The HAICCR group demonstrated significantly higher 1-year, 2-year, and 3-year OS rates of 56.8%, 18.8%, and 4.5%, respectively, compared to the rates of 41.2%, 21.8%, and 1.7% (*p* < 0.001) in the CR group. Similarly, the HAICCR group showed significantly higher 6-month, 12-month, and 18-month PFS rates of 63.7%, 34.9%, and 19.2%, compared to the PFS rates of 38.7%, 16.0%, and 6.7% (*p* < 0.001) in the CR group. In the matched cohort, the OS and PFS of the triple regimen group were significantly longer than those of the dual regimen group (median OS: 18.7 months (HR: 0.93, 95% CI 16.88–20.52) vs 11.0 months (HR: 1.93, 95% CI 7.20–14.74), *p* < 0.0001; median PFS: 10.0 months (HR: 1.19, 95% CI 7.68–12.32) vs 5.6 months (HR: 0.74, 95% CI 4.14–7.06), *p* < 0.0001). Furthermore, the HAICCR group demonstrated superior 1-year, 2-year, and 3-year OS rates of 55.4%, 18.1%, and 4.8%, compared to the rates of 41.2%, 21.8%, and 1.7% in the CR group. The 6-month, 12-month, and 18-month PFS rates for the HAICCR group were 67.5%, 33.7%, and 18.1%, respectively, while the rates of the CR group were 38.6%, 14.5%, and 7.2%, and the HAICCR group exhibited a longer duration (*p* < 0.0001). Kaplan–Meier curves of the HAICCR group and the CR group are shown in Fig. 2. Forest plot analysis of factors associated with OS and PFS in subgroup analysis can be seen in Fig. 3. In general, the HAICCR group renders easier tumor progression control and clinical advantages in patients exhibiting PS 1, tumor diameter > 7 cm, and non-extrahepatic metastases as opposed to the CR group.

### Tumor response

According to the mRECIST 1.1 criteria, in the overall cohort, the triple therapy group had 15 cases achieving CR, 147 cases achieving PR, 98 cases assessed as SD, and 32 cases showing PD, whereas in the dual therapy group, 5 cases achieved CR, 45 cases achieved PR, 44 cases achieved SD, and 25 cases showed PD. In terms of overall treatment response, the HAICCR group was more advantageous than the CR group (*p* = 0.024). The HAICCR group had a higher ORR and DCR at 55.5% and 89.0% respectively, compared to the CR group at 42.0% and 79.0%. After matching, the triple therapy group showed 1 case achieving CR, 34 cases achieving PR, 43 cases assessed as SD, and 5 cases showing PD, while the dual therapy group showed 5 cases achieving CR, 30 cases achieving PR, 30 cases assessed as SD, and 18 cases showing PD. The former group was superior (*p* = 0.006). Both groups exhibited a comparable ORR of 42.2%, with no significant difference observed. Notably, the DCR in the HAICCR group was notably higher at 94.0% when compared to the CR group’s DCR of 78.3%. Treatment responses are shown in Table 2.

**Table 2:** Best tumor response before and after the adjustment of PSM

	Before PSM			After PSM		
	HAICCR group (*n* = 292)	CR group (*n* = 119)	*p* value	HAICCR group (*n* = 83)	CR group (*n* = 83)	*p* value
Best response			0.024			0.006
CR	15 (5.1)	5 (4.2)		1 (1.2)	5 (6.0)	
PR	147 (50.3)	45 (37.8)		34 (41.0)	30 (36.1)	
SD	98 (33.6)	44 (37.0)		43 (51.8)	30 (36.1)	
PD	32 (11.0)	25 (21.0)		5 (6.0)	18 (21.7)	
ORR	55.5% (162/292)	42.0% (50/119)	0.013	42.2% (35/83)	42.2% (35/83)	1.000
DCR	89.0% (260/292)	79.0% (94/119)	0.008	94.0% (78/83)	78.3% (65/83)	0.007

^*^Data are presented as *n* (%)

*PSM* propensity score matching; *HAICCR* hepatic artery infusion chemotherapy plus camrelizumab and rivoceranib, *CR* camrelizumab combined with rivoceranib, *CR* complete response, *PR* partial response, *SD* stable disease, *PD* progressive disease, *ORR* objective response rate, *DCR* disease control rate

### Factors contributing to survival outcomes

The influence factors of OS and PFS in the univariate and multivariate analysis are shown in Table 3. The COX proportional hazards regression model was utilized for both univariate and multivariate analyses. Prognostic factors with a *P* value < 0.1 in the univariate analysis were selected for inclusion in the multivariate analysis. Ultimately, CTP stage A, ALBI grade 1, tumor number ≤ 3, and receiving the HAICCR triple therapy regimen were shown as independent prognostic factors affecting OS. AFP level ≤ 400 μg/L, tumor numbers one to three, presence of metastasis, and receiving the HAICCR triple therapy regimen were found to be prognostic factors associated with PFS.

**Table 3:** Risk factors for overall survival and progression-free survival based on uni- and multivariate analysis

Factors	Overall survival				Progression-free survival			
	Univariate analysis *p* value	Multivariate analysis			Univariate analysis *p* value	Multivariate analysis		
		HR	95% CI	*p* value		HR	95 % CI	*p* value
Gender	0.446	–	–	–	0.120	–	–	–
Male								
Female								
Age	0.827	–	–	–	0.741	-	-	-
≤ 65 y								
> 65 y								
ECOG	0.582	–	–	–	0.191	–	–	–
0								
1								
HBV	0.770	–	–	–	0.826	–	–	–
Presence								
Absence								
CTP stage	< 0.0001	1.872	1.140–3.074	**0.013**	0.202	–	–	–
A								
B								
ALBI grade	< 0.0001	1.831	1.297–2.584	**0.001**	0.119	–	–	–
1								
2								
AFP	0.278	–	–	–	0.084	1.477	1.144–1.907	**0.003**
≤ 400 ng/L								
> 400 ng/L								
Tumor size	0.425	–	–	–	0.628	–	–	–
≤ 7 cm								
> 7 cm								
Tumor number	< 0.0001	2.093	1.546–2.834	** < 0.0001**	< 0.0001	1.808	1.396–2.343	** < 0.0001**
1–3								
> 3								
Metastasis	0.016	–	–	–	< 0.0001	1.328	1.042–1.694	**0.022**
Presence								
Absence								
Treatment regime	< 0.0001	2.082	1.551–2.795	** < 0.0001**	< 0.0001	2.100	1.588–2.776	** < 0.0001**
H-C-R								
C-R								

*HR* hazard ratios, *CI* confidence interval, *ECOG* Eastern Cooperative Oncology Group Score, *HBV* hepatitis B virus, *CTP* Child–Turcotte–Pugh, *AFP* α-fetoprotein

Bold indicates statistical significance level at *p* value < 0.05

### Safety

The occurrence of grade 1 or 2 TRAEs and grade 3 or 4 TRAEs exhibited comparability between the group receiving HAIC–camrelizumab–rivoceranib treatment and the group receiving camrelizumab–rivoceranib treatment, as indicated in Table 4. A total of 263 out of 292 patients (263/292, 90.1%) experienced TRAEs in the HAICCR group, whereas 96 out of 119 patients (96/119, 80.7%) experienced TRAEs in the camrelizumab–rivoceranib group. The triple therapy group exhibited hypertension (44.8%), fever (18.1%), and hand–foot syndrome (17.8%) as the most prevalent grade 3 or 4 TRAEs. Conversely, hypertension (39.4%), hand–foot syndrome (14.3%), and elevated gamma-glutamyltransferase levels (12.3%) were the most frequently observed grade 3 or 4 TRAEs in dual therapy (camrelizumab–rivoceranib). Among the patients in the experimental group, no discontinuation of HAIC was attributed to AEs, but therapy interruption or dose reduction of HAIC could be observed in 90 of 292 patients. 15 and 42 cases resulted in camrelizumab discontinuation and rivoceranib discontinuation, respectively, due to AEs. All treatment medications were discontinued in ten cases due to adverse reactions. Rivoceranib interruption and dose reduction in relation to adverse reactions were observed in 155 cases. Four cases (4/119, 3.4%) of camrelizumab discontinuation, 11 cases (11/119, 9.2%) of rivoceranib discontinuation, 2 cases (2/119, 1.7%) of discontinuation of all treatment medications, and 51 cases of rivoceranib interruption and dose reduction owing to the aforementioned reasons were recorded in 119 patients in the CR group. The incidence of any grade of complication-related deaths was not reported in the overall cohort.

**Table 4:** Treatment-related adverse events

Adverse events	Grade 1/2				Grade 3/4		
	HAICCR (*n* = 292)	CR (*n* = 119)	*p* value		HAICCR (*n* = 292)	CR (*n* = 119)	*p* value
Any adverse events	57 (19.5)	22 (18.5)	0.918		206 (70.5)	74 (62.2)	0.125
AEs-related treatment interruption or dose reduction							
HAIC discontinuation	0 (0%)	NA	-		0 (0%)	NA	-
Camrelizumab discontinuation	4 (1.4)	1 (0.8)	1.000		11 (3.8)	3 (2.5)	0.740
Camrelizumab–rivoceranib discontinuation	3 (1.0)	0 (0)	0.638		7 (2.4)	2 (1.7)	0.937
Rivoceranib discontinuation	9 (3.1)	2 (1.7)	0.644		33 (11.3)	9 (7.6)	0.339
HAIC interruption and dose reduction	34 (11.6)	NA	-		56 (19.2)	NA	-
Rivoceranib interruption and dose reduction	64 (21.9)	17 (14.2)	0.104		91 (31.2)	33 (27.7)	0.569
Treatment-related AEs							
Hypertension	93 (31.8)	34 (28.6)	0.593		131 (44.8)	47 (39.4)	0.969
Fatigue	58 (19.9)	22 (18.5)	0.855		6 (2.1)	2 (1.7)	0.670
Diarrhea	69 (23.6)	37 (31.1)	0.149		2 (0.7)	0 (0)	0.902
Inappetence	61 (20.9)	24 (20.2)	0.976		8 (2.7)	3 (2.5)	1.000
Headache	51 (17.5)	18 (15.1)	0.667		6 (2.1)	1 (0.8)	1.000
Fever	105 (36.0)	47 (39.5)	0.575		53 (18.1)	12 (10.0)	0.060
Abdominal pain	45 (15.4)	13 (10.9)	0.304		2 (0.7)	2 (1.7)	0.705
Gingival bleeding	33 (13.4)	9 (7.6)	0.339		1 (0.3)	0 (0)	1.000
Neurologic toxicity	19 (6.5)	3 (2.5)	0.116		0 (0)	0 (0)	1.000
Hypothyroidism	19 (6.5)	7 (5.9)	0.990		1 (0.3)	0 (0)	1.000
Hyperthyroidism	15 (5.1)	4 (3.4)	0.604		0 (0)	0 (0)	1.000
Pneumonitis	28 (9.6)	10 (8.4)	0.850		1 (0.3)	0 (0)	1.000
Cough	77 (26.4)	32 (26.9)	1.000		3 (1.0)	1 (0.8)	1.000
Dyspnea	3 (1.0)	2 (1.7)	0.959		3 (1.0)	0 (0)	0.638
Rash	55 (18.8)	19 (16.0)	0.586		5 (1.7)	2 (1.7)	1.000
Hand–foot syndrome	94 (32.2)	31 (26.1)	0.267		52 (17.8)	17 (14.3)	0.471
Diarrhea	83 (28.4)	39 (32.8)	0.450		4 (1.4)	1 (0.8)	1.000
Vomiting	86 (29.5)	30 (25.2)	0.456		7 (2.4)	2 (1.7)	0.937
Nausea	94 (32.2)	28 (23.5)	0.104		4 (1.4)	0 (0)	0.466
Laboratory-related AEs							
Anemia	81 (27.7)	25 (21.0)	0.197		4 (1.3)	1 (0.8)	1.000
Leukopenia	103 (35.3)	32 (26.9)	0.127		40 (13.6)	15 (12.6)	0.892
Neutropenia	109 (37.3)	39 (32.8)	0.448		32 (11.0)	13 (10.9)	0.921
Thrombocytopenia	89 (30.5)	35 (29.4)	0.924		29 (9.9)	10 (8.4)	0.769
Elevated ALT	128 (43.8)	49 (38.6)	0.393		34 (11.6)	11 (9.2)	0.594
Elevated AST	134 (45.9)	51 (42.8)	0.652		30 (10.2)	9 (7.5)	0.506
Gamma-glutamyltransferase increase	62 (21.2)	21 (17.6)	0.209		36 (12.3)	13 (10.9)	0.618
Hypophosphatemia	18 (6.1)	5 (4.2)	0.583		10 (3.4)	2 (1.7)	0.529
Hypoalbuminemia	54 (18.4)	18 (15.1)	0.556		2 (0.7)	1 (0.8)	1.000
Hyperbilirubinemia	95 (32.5)	41 (34.5)	0.502		6 (2.1%)	4 (3.4)	0.670
Elevated creatinine	91 (31.2)	33 (27.7)	0.569		7 (2.4%)	2 (1.7)	0.937
Proteinuria	97 (33.2)	37 (31.1)	0.763		19 (6.5)	6 (5.0)	0.737

Data represented as: *n *(%)

*AEs* adverse events, *HAIC* hepatic artery infusion chemotherapy, *HAICCR* hepatic artery infusion chemotherapy plus camrelizumab and rivoceranib, *CR* camrelizumab combined with rivoceranib

## Discussion

To the best of our knowledge, this is the first study to explore the effectiveness and safety of the combination strategy of mFOLFOX6-HAIC and camrelizumab plus rivoceranib in treating HCC with PVTT. In this multicenter retrospective study, HCC patients with PVTT who received triple therapeutic regimen of HAIC combined with camrelizumab and rivoceranib achieved remarkable median OS. The addition of HAIC to molecular tyrosine kinase inhibitor (TKI) plus anti-PD-1 therapy also helped further improvement in terms of PFS, ORR, and DCR. Consistent survival outcomes could be observed after propensity matching except advantages in ORR. Grade 3 or 4 TRAEs more frequently occurred in the triple combination regime (70.5% vs 62.2%), but without statistical differences between groups. In addition, multivariate analysis revealed that the triple combination strategy was identified as an independent factor influencing OS and PFS according to multivariate analysis.

PVTT constitutes a significant influencing factor in the unfavorable prognosis of HCC, whereas there still remains controversy about standard treatment methods. Surgical resection is one of the preferred treatment options for PVTT, but only a minority of patients are evaluated as suitable candidates (Child–Pugh A and typeI/II PVTT) [25]. The combined approach of TACE with sorafenib or radiotherapy has shown promising outcomes; however, it remains a formidable challenge to effectively manage tumor progression [26, 27]. Moreover, there is also an obvious limitation of the capability of BCLC-recommended anti-angiogenic therapy alone in the management of advanced HCC with PVTT. Besides, the updated IMbrave150 study failed to achieve positive results and reveals that the median OS for the combination therapy of bevacizumab and atezolizumab in treating PVTT is only 7.6 months [8]. Currently, the RESCUE phase II trial [15] and CARES-310 phase III trial [16], encompassing a substantial proportion of patients with PVTT, aimed to compare the efficacy of camrelizumab plus rivoceranib with sorafenib, and both achieved positive results in first-line treatment of advanced HCC. Up to now, the application of camrelizumab plus rivoceranib in HCC with PVTT was only reported in a multicenter retrospective study, with an mOS of 14.8 months, which significantly further extended around 9–10 months of mOS on the basis of sorafenib [17]. By contrast, the results in our study are less superior, and the relatively unfavorable prognosis in terms of mOS is mainly because of the higher tumor burden and a higher proportion of metastasis in the patients included in this study, which makes it more difficult to shrink intrahepatic tumor lesions.

In this study, the triplet therapy group showed significant superiority over the dual therapy group in terms of OS and PFS both before and after PSM. After adjusting for data balance with PSM, the median OS and median PFS for the dual therapy group were only 11.0 and 5.6 months, respectively, while the triplet therapy group had an OS of 18.7 months and a PFS of 10.0 months, resulting in an extension of nearly 8 months in median OS compared to the former. On the other hand, the overall tumor response, ORR, and DCR of the HAICCR group in the entire cohort were significantly higher than those of the CR group. However, in the propensity score-matched cohort, there was no statistical difference in ORR between the groups. Interestingly, prolonged survival often resulted from a higher ORR in some previous studies [28]. Remarkably, in this study, despite not achieving a higher ORR after balancing the data, there was a significant extension in OS and PFS. Indeed, unbiased data after PSM adjustment indicates that, based on the mRECIST 1.1 criteria, the main difference between the HAICCR group and the CR group lies in the higher proportion of SD in the HAICCR group. Previous studies have confirmed that the subgroup of HCC with portal vein involvement demonstrates rapid and high-risk intrahepatic and extrahepatic progression. Although the HAICCR triple therapy did not show superior tumor shrinkage effects, its role in stabilizing disease lesion within this high-risk subgroup appears evident. For the CR group, experiencing a higher proportion of PD likely contributes to poorer prognosis due to the direct impact of intrahepatic and extrahepatic progression. Therefore, maintaining disease stability may be the main reason for the HAICCR group’s longer OS and PFS.

Such remarkable survival outcomes may be attributed to the reciprocal regulatory reaction and synergistic effect of chemotherapy agents, anti-angiogenic therapy, and the blockade of the PD-1/PD-L1 pathway. On the one hand, the inhibitors of VEGFR can not only facilitate the restructuring of tumor blood vessels, promoting vascular normalization and consequently enhancing the efficacy and resistance to chemotherapy and anti-PD-1/PD-L1 treatment [29], but also exhibit the capability to suppress the immune-inhibitory myeloid cell recruitment induced by VEGF-A. This inhibition effectively counteracts the immunosuppressive effects exerted by aberrant myeloid populations and improves the tumor immune microenvironment, thus enhancing the resistance to immunotherapy [30, 31]. On the other hand, within the mFOLFOX regimen-based HAIC, oxaliplatin assumes a crucial role by facilitating immunogenic cell death, thus promoting immune activation and augmenting the antigenicity of tumor cells in the tumor cell microcirculation. Besides, immunogenic cell death can also improve the antigenicity of tumors to sensitize “off-target” cells in the tumor microenvironment to inhibit the proliferation and migration of tumor cells [32–36]. As a result, these three factors collectively exhibit a synergistic effect and, to a certain extent, compensate for their individual deficiencies, for fully unleashing their anti-tumor effects.

In this study, multivariate analyses identified CTP stage A, ALBI grade 1, tumor number ≤ 3, and treatment regime as risk factors influencing OS, while AFP level, tumor number, metastasis, and treatment regime were considered as risk factors affecting PFS. Among them, CTP stage and ALBI grade are established criteria for assessing liver function, and ALBI grading eliminates the subjective impact of ascites and hepatic encephalopathy, making it a more objective reference standard. Additionally, the existing medical evidence has already confirmed that AFP dynamically reflects tumor activity and indirectly reflects the tumor progression capability. For subgroup comparison, the triple combination regime showed effective management in HCC patients with high tumor burden. Patients aged ≤ 65 years old, ECOG PS 0, tumor size > 7 cm, with HBV infection and metastasis were more suitable to add HAIC to the administration of camrelizumab and rivoceranib for profitable OS and PFS, but age, ECOG PS 0, and HBV infection-related ineffectiveness of the triple combination regime may be primarily due to insufficient sample size within these subgroups.

In the context of liver function impairment as a main TRAE, the HAICCR group exhibited a slightly higher incidence than the CR group, albeit without significant statistical difference. Furthermore, certain adverse reactions such as hematuria, cough, diarrhea, fever, and hyperbilirubinemia have a higher incidence in the CR group, and the reasons for these differential occurrences are largely attributed to the biases inherent in the retrospective study design. At baseline, the CR group exhibited relatively poorer baseline conditions, with a higher proportion of metastases and impaired organ function, thereby rendering them more sensitive to drug-related adverse events. Therefore, in such circumstances, adverse events in the overall CR group may have been relatively overestimated, leading to a reduction in the observed differences between the HAICCR group and the CR group.

This study still has some inherent limitations that should be acknowledged. Firstly, due to its retrospective nature, it inherently introduces unavoidable selection bias. While we employed propensity score matching to minimize between-group differences, endogenous differences may still exist. Thus, there is a need for additional prospective randomized controlled trials to enhance the level of evidence and further validate our findings. Secondly, the relatively small sample size in certain subgroups underscores the importance of expanding the study population and conducting larger-scale research to enhance the reliability of the findings. Then, this study incorporated multicenter data, thereby potentially introducing variations in treatment approaches across different centers that could impact the findings of this study. Finally, it is noteworthy that a significant portion of patients encompassed in this study exhibited viral hepatitis, engendering potential incongruity in the comprehensive demographic of globally encompassed unresectable HCC patients. Hence, further validation through larger international trials is warranted.

## Conclusion

The integration of HAIC into the camrelizumab–rivoceranib regimen has showcased enhanced efficacy and well-tolerated adverse reactions for HCC with PVTT. This amalgamation of therapies harbors encouraging prospects as a feasible choice for HCC with PVTT.

## Data Availability

The data underpinning the discoveries of this study can be accessed from the corresponding author upon inquiry, in compliance with reasonable stipulations.
